# Supplementary material for: Electrostatic Charge Retention in PVDF Nanofiber-Nylon Mesh Multilayer Structure for Effective Fine Particulate Matter Filtration for Face Masks
Source: Polymers (Basel). 2021 Sep 24;13(19):3235. doi: 10.3390/polym13193235 (PMC8513023; doi:10.3390/polym13193235)
Supplement: Supplementary file 1 [file polymers-13-03235-s001.zip › polymers-1359955-supplementary.pdf]

SUPPLEMENTARY MATERIAL

Electrostatic Charge Retention in PVDF Nanofiber-Nylon Mesh Multilayer Structure for Effective Fine Particulate Matter Filtration for Face Masks

Dong Hee Kang, Na Kyong Kim and Hyun Wook Kang *

Department of Mechanical Engineering, Chonnam National University

77 Youngbong-ro, Buk-Gu, Gwangju 61186, Republic of Korea

August, 2021

*Corresponding author: Prof. Hyun Wook Kang

Tel: +82-62-530-1662

Fax: +82-62-560-1689

E-mail: kanghw@chonnam.ac.kr

Supplementary Material

Table S1. Comparison of absolute humidity of normal breathing of adults with continuous air flow in the apparatus to control the absolute humidity.

Table S2. Power of airflow to bending filter layer during normal breathing condition and cyclic air blowing condition.

Table S3. Density thickness of the PVDF bulk and NF membrane film for porosity calculation.

Table S4. Characteristics of the commercial disposable face mask filter via pressure difference, filtration efficiency, and QF analysis.

Figure S1. Pressure drop of the nylon mesh supporting layer depending on the number of stacking layers.

Figure S2. Pneumatic symbol of the experimental set up for PM filtration test.

Figure S3. Pneumatic symbol of the humid air supply set up for the filter discharging.

Figure S4. Image of the apparatus for electrostatic charging of the multi-layer filter by a cyclic air blower.

Figure S5. Schematics and diameter distributions of the 13 wt% electrospun PVDF nanofiber (a, b) without and (c, d) with nylon mesh on the Al foil collector.

Figure S6. Correlation of the electrospinning time for PVDF NF membrane fabrication and its thickness.

Figure S7. QF of single layer filter due to the presence of electrostatic charges with the NF membrane fabrication time according to PM size.

Figure S8. Pressure drop of the multilayer filter as a function of number of stacking layers after dry or humid air penetration for 0.5 h.

Supplementary Material

Table S1. Comparison of absolute humidity of normal breathing of adults with continuous air flow in the apparatus to control the absolute humidity.

	Average absolute humidity for normal breathing of adult	Humid air supply setup
Absolute humidity [mg L^{-1}]	30.4[1]	15.56*
Flow rate per minute [L min^{-1}]	6~10**[2,3]	22.5***
Average absolute humidity per minute [mg min^{-1}]	182.4 ~ 304.0	350.1

* Calculated absolute humidity from psychrometric chart based on the measured value from the temperature humidity sensor in the upstream filter chamber (for 20 °C under 90% relative humidity).

** Flow rate of normal adult breathing: Tidal volume in normal adult [2] (0.5 L min^{-1} an exhaled breath) and breath per minute in normal adults [3] (12-20 breathing per minute).

*** A flow rate of vacuum pump in the humid air supply apparatus.

Table S2. Power of airflow to bending filter layer during normal breathing condition and cyclic air blowing condition.

	Airflow during normal breathing	Airflow via apparatus for cyclic air blowing
Flow rate $\cdot 10^{-3}$ [m^3 s^{-1}]	0.25*[4,5]	0.283***
Pressure difference [Pa]	400**[6]	180
Power of the airflow for filter bending [W]	0.10	0.05

* Maximum flowrate of exhaled breath in the normal state of adults is calculated under conditions of tidal volume change [4] in a normal breathing cycle (15 breaths per minute). [5]

** Pressure difference between the atmospheric pressure and the intrapulmonary pressure. [6]

*** Flow rate is calculated by average velocity of airflow (10 m s^{-1} at 50 mm distance from the end of the air hose tip) and sectional area of the air hose (Diameter: 6 mm).

The porosity (φ) of the film is expressed by Equation (S1).

$$\varphi(\%) = \left(1 - \frac{w_{membr}}{\rho \cdot A \cdot t}\right) \cdot 100 \qquad \text{(S1)}$$

where w_{membr} is the weight of the PVDF NF membrane which is performed electrospinning on 70 mm hole acrylic plate-covered Al foil surface for 17.5 min and cut into 20 × 20 mm^2. The NF membrane thickness was controlled to the same thickness as that of the bulk film for porosity analysis. ρ is the density of the pristine PVDF material, and A and t are the area and thickness of the bulk PVDF film, respectively. Based on the bulk PVDF film thickness, the electrospinning time was controlled to fabricate the bulk film with the same volume. As summarized in Table S3, there is no difference between the experimental and theoretical density thicknesses of the bulk PVDF film, where the film is packed homogeneously without defects or bubbles in its volume. The calculated porosity of the PVDF NF membrane is 92.76%; conventional nanofibrous membranes exhibit a similar porosity of approximately 90%. [7]

Table S3. Density thickness of the PVDF bulk and NF membrane film for porosity calculation.

PVDF Film	Bulk (Experimental)	Bulk* (Theoretical)	NF membrane
Density thickness ($g\ m^{-2}$)	81.667	80.288	3.449

*Theoretical results of bulk film density thickness are calculated by the PVDF density (1,780 kg/m^3) and average thickness (μm) of a bulk film.

Table S4. Characteristics of the commercial disposable face mask filter via pressure difference, filtration efficiency, and QF analysis.

Disposable face mask	ΔP (Pa)	$\eta_{0.3}$ (%)	$\eta_{0.5}$ (%)	$QF_{0.3}$ (Pa^{-1})	$QF_{0.5}$ (Pa^{-1})
Dental mask-1	109 ± 2.0	40.9	50.4	0.0048	0.0064
Dental mask-2	67 ± 1.5	82.8	84.1	0.0265	0.0277
KF 80 grade mask*	112 ± 2.0	90.9	92.0	0.0214	0.0225
KF 94 grade mask*	151 ± 2.5	99.7	99.7	0.0396	0.0395

ΔP (Pa): Pressure drop at 18.64 cm/s, η (%): Filtration efficiency of PM 0.3 and 0.5, QF (Pa^{-1}): $-\ln(1-\eta)/\Delta P$

* The Korea Filter (KF) 80 and 94 grades are based on the protocol for disposable face masks from the Korean Ministry of the Food and Drug Safety (MFDS); these masks have over 80% and 94% efficiency in filtration tests, respectively, using sodium chloride particles and paraffin oil aerosols.

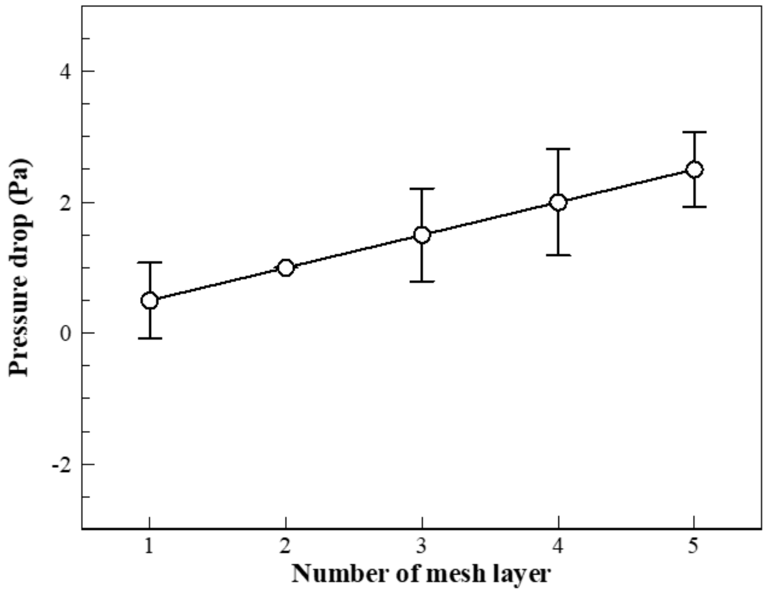

Figure S1. Pressure drop of the nylon mesh supporting layer depending on the number of stacking layers.

To measure the filtration performance, all the single and multilayer filter samples were discharged to create the intrinsic electrostatic condition of each filter, as shown in Figure S2. The remaining charges on the filter surfaces were removed by ethanol dipping, and the surfaces were dried sufficiently at 40 °C for 12 h using a convection oven. To charge the surface under the same conditions, surface charging was performed through bending-release friction 200 times for 90 s at a 2.22 Hz cycle.

As shown in the pneumatic symbols for the filter test apparatus setup, PM particles are generated by incense burning in the 1st dilution tank and they flow by pressure difference for vacuum pump at the end of the apparatus. The PM concentration was controlled by passing through a globe valve and 2nd dilution tank. The filtration efficiency was measured by the number of particles from the upstream and downstream of the filtration section using PM counters (DT-9881m, CEM Inst Co.).

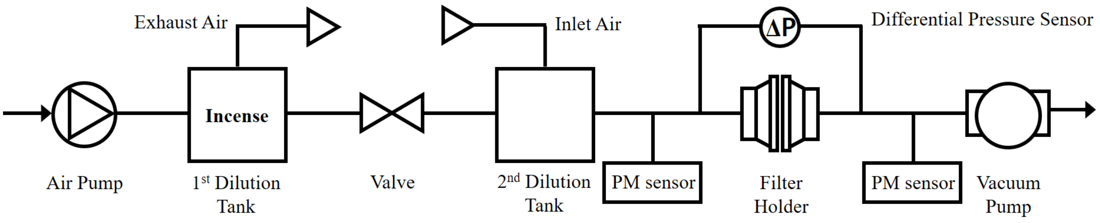

Figure S2. Pneumatic symbol of the experimental set up for PM filtration test.

To obtain the optimum condition for the discharge characteristics of the filter via humid air exposure in continuous flow, the chamber setup was configured as shown in Figure S3. The humid air passing through the filter in the chamber was adjusted to contain the absolute moisture of the exhaled breath from normal adults (Table S1). Large liquid droplets in the flow from the humidifier in the 1st dilution chamber were filtered out in the 2nd dilution chamber and releasing the wheel of the globe valve. The humidity difference was measured using humidity sensors (DHT11) installed upstream and downstream of the filtration section.

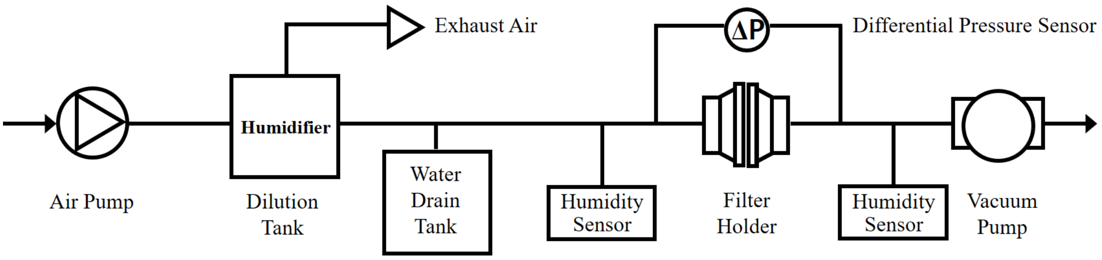

Figure S3. Pneumatic symbol of the humid air supply set up for the filter discharging.

In the apparatus for cyclic air blowing, an air compressor was used for filter deformation to continuously supply air. The flow rate with air pressure and flow direction were controlled using a ball valve and a solenoid valve, respectively. As shown in Figure S4, the tips of the two air hoses are clamped facing each other and are 100 mm apart, and the filter layer is positioned between them. The average air velocity was measured using a hot-wire anemometer (TES-1341, TES Electrical Electronic Corp.) placed between the air hose tips. The filter holder distance was adjusted to loosen the filter so that it could be sufficiently bent and fluttered via cyclic air blowing. The maximum amplitude of the filter deformation was restricted to 10 mm. Cyclic air blowing was performed by changing the flow direction 15 times per minute for 10 min, as summarized in Table S2.

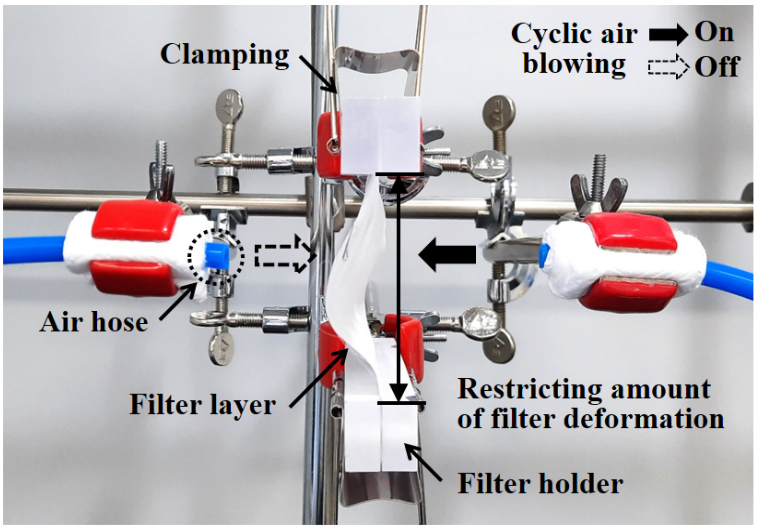

Figure S4. Image of the apparatus for electrostatic charging of the multi-layer filter by a cyclic air blower.

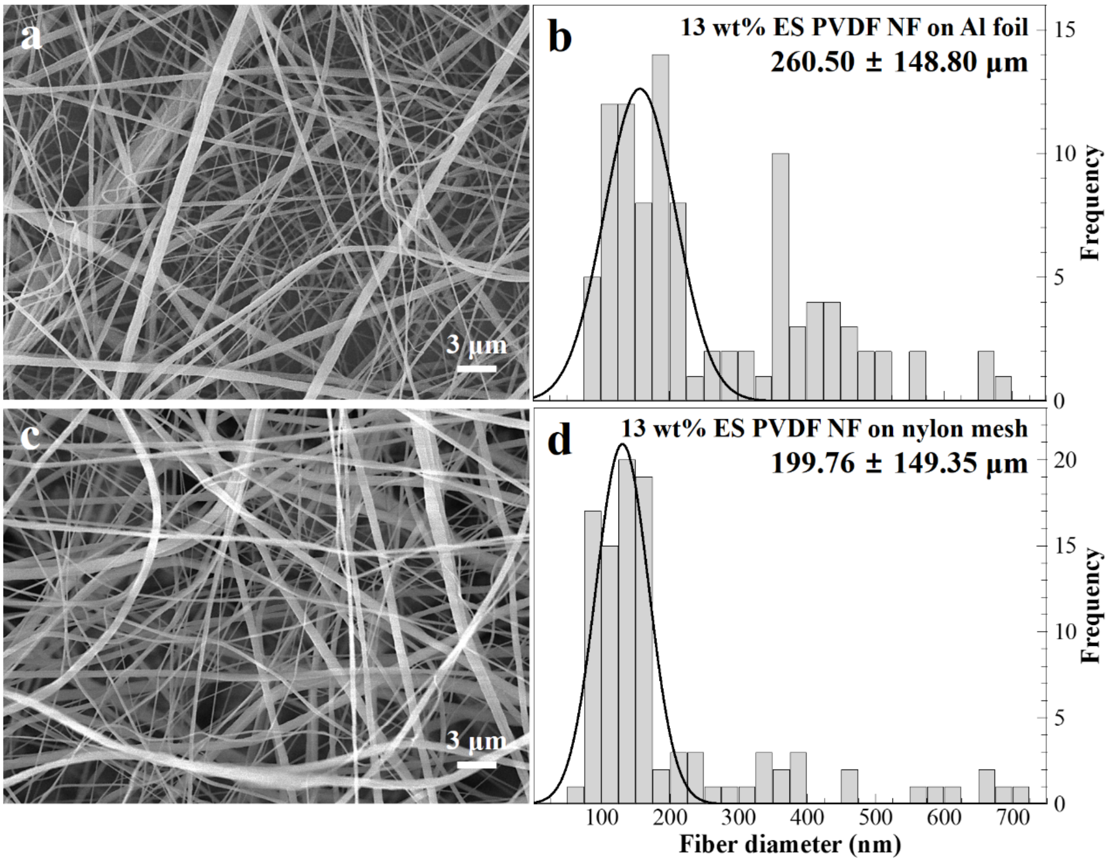

Figure S5. Schematics and diameter distributions of the 13 wt% electrospun PVDF nanofiber (a, b) without and (c, d) with nylon mesh on the Al foil collector.

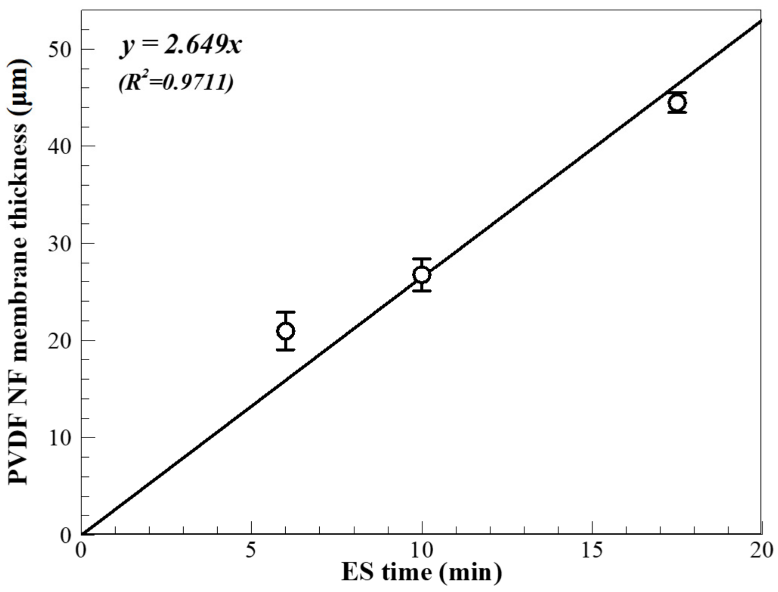

Figure S6. Correlation of the electrospinning time for PVDF NF membrane fabrication and its thickness.

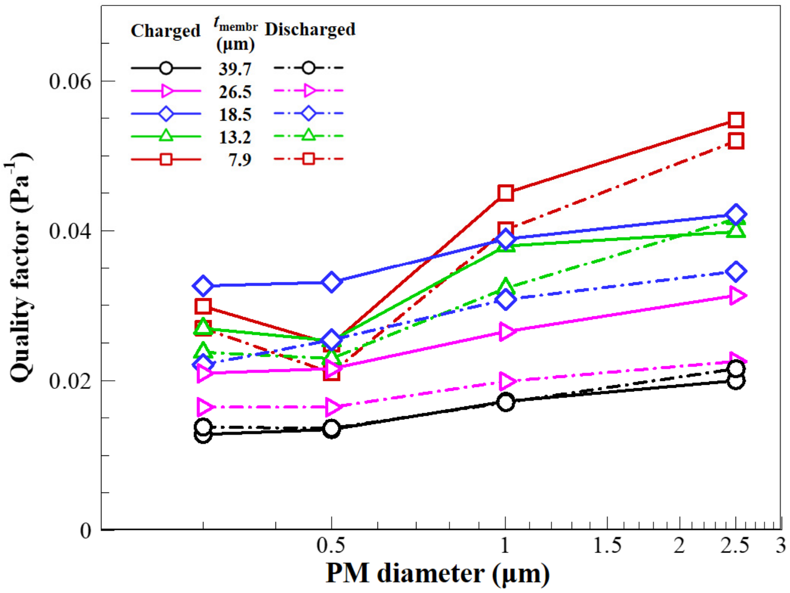

Figure S7. QF of single layer filter due to the presence of electrostatic charges with the NF membrane fabrication time according to PM size.

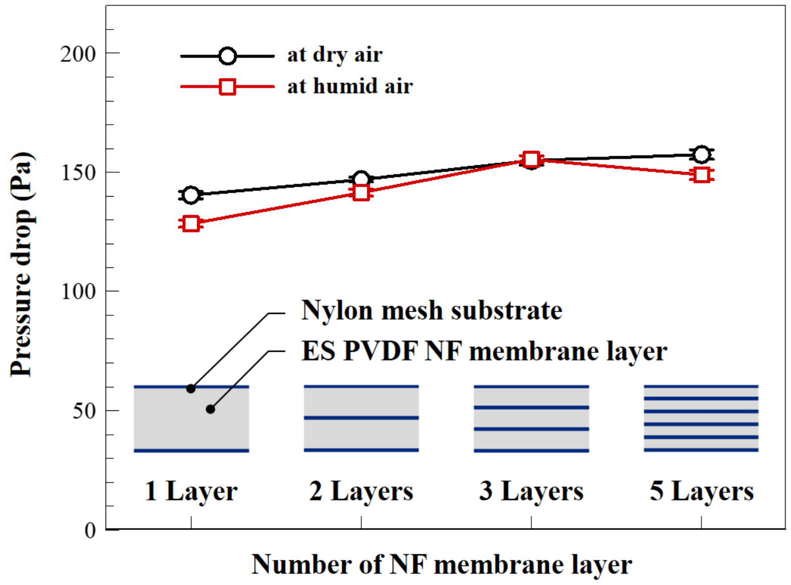

Figure S8. Pressure drop of the multilayer filter as a function of number of stacking layers after dry or humid air penetration for 0.5 h.

References

[1] McFadden Jr, E. R., Pichurko, B. M., Bowman, H. F., Ingenito, E., Burns, S., Dowling, N. & Solway, J. (1985). Thermal mapping of the airways in humans. Journal of applied physiology, 58(2), 564-570.

[2] Tortora, Gerard J. (2016). Principles of anatomy & physiology. Derrickson, Bryan (15th ed.). Hoboken, NJ. p. 874. ISBN 978-1119447979. OCLC 1020568457.

[3] Wilburta Q. Lindh; Marilyn Pooler; Carol Tamparo; Barbara M. Dahl (9 March 2009). Delmar's Comprehensive Medical Assisting: Administrative and Clinical Competencies. Cengage Learning. p. 573. ISBN 978-1-4354-1914-8.

[4] Hulbert, D. (Ed.). (2009). Get Through MCEM Part A: MCQs. CRC Press, p.33.

[5] Walker, H. K., Hall, W. D., & Hurst, J. W. (Eds.). (1990). Clinical methods: the history, physical, and laboratory examinations. Butterworth-heinemann. (3rd ed., PMID: 21250045., Chapter 43. Respiratory Rate and Pattern)

[6] Boone, T. (2014). Introduction to exercise physiology. Jones & Bartlett Publishers. (Chapter 2. Pulmonary Ventilation)

[7] Xiong, J., Zhou, M., Zhang, H., Quan, Z., Wang, R., & Qin, X. (2018). Sandwich-structured fibrous membranes with low filtration resistance for effective PM2. 5 capture via one-step needleless electrospinning. Materials Research Express, 6(3), 035027.
